# Effects of *Isaria fumosorosea* on *TYLCV* (*Tomato Yellow Leaf Curl Virus*) Accumulation and Transmitting Capacity of *Bemisia tabaci*

**DOI:** 10.1371/journal.pone.0164356

**Published:** 2016-10-07

**Authors:** Bowen Zhang, Chunhua Zou, Qiongbo Hu

**Affiliations:** Key Laboratory of Bio-Pesticide Innovation and Application of Guangdong Province, College of Agriculture, South China Agricultural University, Guangzhou, 510642, China; Stony Brook University, UNITED STATES

## Abstract

*Tomato yellow leaf curl virus* (*TYLCV*) is transmitted by the *Bemisia tabaci* pest Middle East-Asia Minor 1 (MEAM1) in China. *Isaria fumosorosea* is a fungal pathogen of *B*. *tabaci*. However, the effects of fungal infection on *TYLCV* expression and transmission by MEAM1 are unclear. In this study, potted tomatoes containing second instar nymphs of MEAM1 were treated with *I*. *fumosorosea* IfB01 strain and the relationship between fungal infection in MEAM1 and its *TYLCV* transmission capacity was investigated. The results indicated that a significantly (p < 0.05) decreased incidence of transmission of *TYLCV*-infected plants (ITYPs) transmitted by second instar nymphs of MEAM1 infected with fungus. Further, we found a negative correlation between fungal conidial concentrations and eclosion rates of MEAM1, and a positive correlation between ITYPs and eclosion. In addition, when each plant was exposed to three adults treated with fungus, a significantly decreased transmission of *TYLCV* (TYTE) was observed in the infected group. However, the incidence of *TYLCV*-carrying MEAM1 adults (ITYAs) was not significantly different in the infected and control groups (p < 0.05). Nevertheless, a significant decrease in viral accumulation using *TYLCV AC2* gene as a marker was observed in the fungus-infected MEAM1. In conclusion, the results suggested that *I*. *fumosorosea* infection decreases *TYLCV* accumulation in MEAM1 and subsequently reduces its transmission. Our study provides new insights into the relationship between host plant, plant virus, insect vector, and entomopathogenic fungus.

## Introduction

*Bemisia tabaci*, is an important insect species comprising harmful pests: Middle East-Asia Minor 1 (MEAM1, i.e., B-biotype) and Mediterranean species (MED, i.e., Q-biotype) [1,2]. *B*. *tabici* transmits the *tomato yellow leaf curl virus* (*TYLCV*), which is a begomovirus infecting plant species, resulting in agricultural damage [1,2]. In China, *TYLCV* was identified in the 1980’s. However, its prevalence increased only after 2006, resulting in serious decrease in tomato production [3,4]. The *TYLCV* epidemic is associated with the invasion and spread of MEAM1 and MED. MEAM1 was detected in China in the early 1990’s, while the MED was found in the 2000’s [5,6]. The relationship between begomoviruses and *B*. *tabaci* is evolutionarily conserved. *TYLCV* is transmitted by *B*. *tabaci* in a disease cycle. Virus survival in the hemolymph of *B*. *tabaci* is ensured by GroEL homologue proteins secreted by a *B*. *tabaci* secondary endosymbiont. Viral spread to non-host plants may lead to association with the insect for its entire 4- to 5-week adult life. During this period, the ability of the insects to inoculate plants is steadily decreased. The long-term presence of *TYLCV*s in *B*. *tabaci* is associated with decline in insect life span and fertility. Viral DNA is transmitted to progeny, without infectivity. *TYLCV* mRNA is detected in insects, suggesting possible replication and expression in the vector. *TYLCV* may spread among *B*. *tabaci* during copulation [7]. However, the pathogenesis and epidemiology of *TYLCV* have yet to be elucidated. Due to the complex interaction between host factors in the plants, viruses, insect vectors and environment, it is essential to understand the mechanisms and patterns of interaction involving “host plant-virus-vector” for integrated pest management.

Chemical insecticides are still the mainstay of *B*. *tabaci* control and management. However, adverse effects including resistance to neo-nicotinoids led to MED outbreak and *TYLCV* prevalence in China [3]. Therefore, *TYLCV* control requires development of new integrated pest management (IPM) technologies against its insect vector, *B*. *tabaci*. Entomogenous fungi mainly invade insects through the cuticle and morphologically transform into blastospores after entering the host hemoceol. To colonize the host insects, the fungus must adapt to the hemolymph and defend against infection by other pathogens [8,9,10,11]. Poisonous fungal entomopathogens represent the best microbes to control sap-sucking pests. In fact, *Beauveria bassiana* [12,13,14], *Metarhizium anisopliae* [15,16], and *Lecanicillium* (*Verticillium*) *lecanii* [17,18], were broadly studied for the biocontrol of *B*. *tabaci*. However, few studies have investigated the interactions from the perspective of “host plant-phytopathogenic virus-insect vector—entomopathogenic fungus”. It is unknown whether fungal entomopathogens infect the host insect transmitting the plant virus.

*Isaria fumosorosea* (*Paecilomyces fumosoroseus*) is an entomopathogenic fungal species that usually infects *B*. *tabaci* nymphs [19]. Several strains of the species were isolated and validated as potential biological control agents in *B*. *tabaci* [20,21,22,23]. In a previous study, we selected the *I*. *fumosorosea* strain, IfB01, which was effective against *B*. *tabaci* [24]. We, therefore, evaluated its role in *TYLCV* transmission of *B*. *tabaci*. This study enhances our understanding of the interaction between host plant, phytopathogenic virus, insect vector, and entomopathogenic fungi.

## Materials and Methods

### Tomato, TYLCV and MEAM1

The Xinjingfeng 1 variety of tomato, *Solanum lycopersicum*, available commercially (Guangzhou Changhe Seeds Company), was used as the host for *TYLCV* and MEAM1. The seeds were planted in 8×6 cm pots and cultured in 60×60×60 cm cages at 25±1°C and exposed to 14h dark/10h light cycle. After germination, only the seedling was maintained in each pot (potted-plant) for further use.

The infectious clone of *TYLCV* was kindly provided by Prof. Zhou Xueping (College of Agriculture and Biotechnology, Zhejiang University, China). A small amount of a *TYLCV* infectious clone was inoculated in 5 mL YEP broth (yeast extracts 10 g/L, peptone 10 g/L, sodium chloride 5 g/L, kanamycin 50 ml/L, streptomycin 50 ml/L and water 1 L) and cultured at 28°C and 200 rpm for 24 h. The broth was collected and subjected to centrifugation at 4000 rpm for 3 min and the supernatant was discarded. The broth containing pellets was transferred to an injector for *TYLCV* inoculation. When the tomato potted-plants grew to the 3-to 4-leaf stage, a 0.2 mL of *TYLCV* inoculum was injected at the stem base of each tomato plant. The plants were further cultured to a 6-7-leaf stage. The plants showing apparent symptoms were selected and subjected to PCR testing to validate the *TYLCV* infection. The *TYLCV*-infected plants (TY-plants) were maintained in 60×60×60 cm cages at 25±1°C and exposed to 14h dark/10h light cycle.

The MEAM1 population was maintained in our lab for more than 20 generations. It was validated by sequencing the PCR fragment of mitochondrial cytochrome oxidase I gene (*mtCOI*) amplified with the consensus primers, C1-J-2195 (5'-TTGATTTTTTGGTCATCCAGAAGT-3') and TL2N-3014 (5'-TCCAATGCACTAATCTGCCATATTA-3')[25]. The MEAM1 insects were fed with pot-grown non-*TYLCV* host, *Brassica alboglabra*, and cultured in 60×60×60 cm cages at 25±1°C and exposed to 14h dark/10h light cycle. The MEAM1 population was maintained in a separate greenhouse and insectarium to prevent contamination from other *B*. *tabaci* and begomovirus.

The basic and interior leaves of the TY-plants were initially covered with a plastic pocket, and two leaves were retained for MEAM1 infestation. Forty MEAM1 adults were introduced from *B*. *alboglabra* plants into each TY-plant. After laying eggs for 1 d, the adults were driven away. When the insects developed to 2^nd^ instar nymphs, their numbers were counted to ensure adequate number in each TY-plant (others were removed).

### Fungal treatment experiment 1

The *I*. *fumosorosea* IfB01 strain (China Center for Type Culture Collection access number: CCTCC M 2012400) was used. The slant was inoculated on a PDA plate and cultured at 26°C for 2 to 3 weeks. The conidia were collected and suspended with 0.02% Tween 80 into a stock solution containing 10^8^ spores/mL. The different working dilutions were prepared from the stock using 0.02% Tween 80 solution.

**Fig 1:**
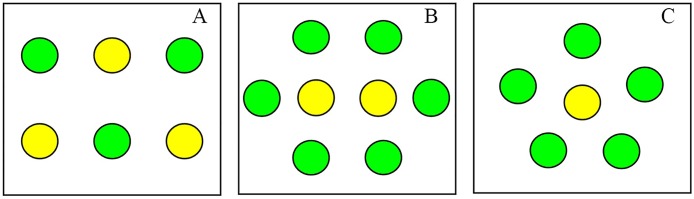
Arrangement of tomato plants in cages in the fungal experiment 1. A: 3 TY-plants (yellow) and 3 normal plants (green) (3/3 cage), B: 2 TY-plants and 6 normal plants (2/6 cage), C: 1 TY-plant and 5 normal plants (1/5 cage).

The fungal treatments were conducted using three different concentrations of IfB01 conidia: 100.0 × 10^6^, 10.0 × 10^6^, and 1.0 × 10^6^ spores/mL. The leaf immersion method (China standard NY/T 1154.14–2008) was used for the bioassay. Before treatment, the potted plants were examined to ensure the presence of adequate number of second instar nymphs of MEAM1. During the treatment, the leaf with MEAM1 nymphs was dipped into each conidial working suspension for 30 s. The control treatment was 0.02% Tween-80. After drying, the potted plants were transferred to cages. Three cages contained three types of plants: 3 TY-plants and 3 normal plants (3/3 cage), 2 TY-plants and 6 normal plants (2/6 cage), 1 TY-plants and 5 normal plants (1/5 cage) (Fig 1). A total of 300 nymphs were allotted to each cage, i.e., 100 nymphs per TY-plant in the 3/3 cage, 150 nymphs for each TY-plant in the 2/6 cage and 300 nymphs targeting each TY-plant in the 1/5 cage (Fig 1). The culture conditions were same as above. All the treatments were performed in triplicate, and the same experiment was repeated twice in 2011–2013 and 2014–2015. The treatments were inspected for insect growth. The nymphs, pupae and adults were counted daily. The eclosion rate was evaluated using the equation: eclosion rate (%) = 100 × puparium number / total nymphs number

The incidence of TY-plants (ITYP) was surveyed on post-eclosion days 3 and 8 after all the pupae molted. The *TYLCV* in each plant was detected as described previously [26]. In brief, leaves weighing up to 150 mg were cut from the top row of normal plants using a clean scissors, and homogenized. The genomic DNA was extracted with DP3111 kit (BioTeke Corporation, Beijing, China) according to the manufacturer’s protocol. The primers *TYLCV*-F (5’-ACGCATGCCTCTAATCCAGTGTA-3’) and *TYLCV*-R (5’CCAATAAGGCGTAAGCGTGTAGAC-3’) were used to PCR amplify the 543 bp target fragment (*TYLCV*SF543) [26]. The PCR mix included 1 μL DNA template, 25 μL 2×MasterMix (BioTeke, PR1701, BioTeke Corporation, Beijing, China), 1 μL of each 10 μmol/L *TYLCV*-F and *TYLCV*-R, and ddH_2_O to obtain a final volume of 50 μL. The PCR conditions were: 94°C pre-denaturation for 3 min, 30 cycles of 94°C denaturation for 30 s, 56°C annealing for 30 s, 72°C extension for 30 s, and a final extension at 72°C for 10 min. PCR products were detected using 1% agarose gel electrophoresis and photography. The plants testing positive were considered as TY-plants. The proportion of TY-plants (ITYP) was evaluated according to the following equation: ITYP (%) = 100 ×TY-plant number / total plant number. The *TYLCV* transmission efficiency of each MEAM1 adult (TYTE) was estimated based on the ITYP values and the number of adults and plants using the following equation: TYTE (%) = ITYP ÷ number of adults ÷ number of plants.

The results were analyzed using analysis of variance (ANOVA), Duncan's multiple range test (DMRT) and regression with SPSS V17.0 statistical software (IBM, USA).

### Fungal treatment experiment 2

The second instar nymphs of MEAM1 generated on TY-plants were treated with an IfB01 conidial suspension of 10×10^6^ spores/mL as described above. After eclosion, the MEAM1 adults were transferred to normal potted plants. Three adults were introduced to each potted plant to transmit *TYLCV* for three days. All the adults were collected separately and stored at -20°C for *TYLCV* tests. Each treatment included 10 potted plants and the experiment was performed in triplicate (30 plants). The ITYPs were investigated similarly after treatment on days 3 and 8 post-treatment.

To evaluate the incidence of *TYLCV*-carrying MEAM1 adults (TY-adults), the *TYLCV* in each adult was detected [27]. Initially, the total DNA was extracted from each adult with an OSR-M401 TGuide Kit (Tiangen Biotech (Beijing) Co., Ltd., Beijing, China) according the manufacturer’s protocol. The *TYLCV*SF543 fragment was amplified with PCR as described above. Thirty adults were tested. The MEAM1 adults testing positive with PCR served as TY-adults. The incidence of TY-adults (ITYA) was evaluated according to the following equation:

ITYA (%) = 100 × TY-adults number / 30, where “30” denotes the total number of MEAM1 adults tested.

The *TYLCV* accumulation in each MEAM1 adult was quantified using qPCR [28]. The PCR primer pairs were TySH-JC-F (5’-GAAACGACCAGTCTGAGGCTGTAATGTC-3’) and TySH-JC-R (5’-AAGAAACCAATAAGGCGTAAGCGTGTAG-3’) to amplify the *AC2* gene (322 bp) fragment derived from *TYLCV*-IL [CN: SH2] genome (AM282874.1). The MEAM1 β-actin gene was used as an internal reference and amplified with the primers β-actin-F (5’-TCACCACCACAGCTGAGAGA-3’) and β-actin-R (5’-CTCGTGGATACCGCAAGATT-3’). The qPCR was performed on a Bio-Rad CFX Connect^™^ Real-time Thermal Cycler (Bio-Rad, USA). The reaction mixture contained 1 μL DNA template for the target genes, 0.5 μL of each primer, 10 μL iTaq^™^ Universal SYBR Green Supermix (Bio-Rad, USA), and 8 μL ddH_2_O. The cycling conditions involved pre-denaturation for 3 min at 94°C, and 40 cycles of denaturation for 30 s at 94°C, annealing for 30 s at 57°C, extension for 30 s at 72°C, and a final extension at 72°C for 10 min. The expression of target genes was quantified based on the values of 2^-ΔΔCt^ [29]. Samples from 17 adults and 18 control adults were analyzed using qPCR.

The results were subject to statistical analysis using Excel 2003 software (Microsoft, USA).

## Results

### Effect of *I*. *fumosorosea* on MEAM1 eclosion

**Fig 2:**
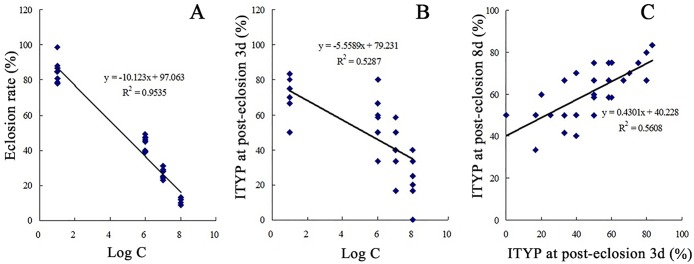
Regression analysis of eclosion rates, logarithm of fungal conidial concentrations (Log C) and ITYP. A: eclosion rate and the logarithm of fungal conidial concentrations; B: ITYP on post-eclosion day 3 and the logarithm of fungal conidial concentrations; C: ITYP on post-eclosion days 3 and 8.

The eclosion rates of MEAM1 were decreased with the fungal conidial dose after the 2^nd^ instar nymphs were treated by *I*. *fumosorosea* IfB01 strain (Table 1). At the fungal conidia concentrations of 100.0, 10.0 and 1.0 (×10^6^ spores/mL), eclosion rates of 11.11%, 25.89% and 43.67% were recorded, respectively. A significantly higher value of 84.89% was observed in the CK (p < 0.05) (Table 1). The ANOVA results indicated significantly different eclosion rates (p < 0.01) of MEAM1 following exposure to varying concentrations of fungal conidia. The differences were insignificant (p > 0.05) when TY-plants were compared with normal plants, and conidia concentrations in TY-plants were compared with normal plants (Table 2). The results suggested that the conidial concentration of IfB01 strain significantly affected the eclosion of MEAM1. However, the comparison of TY-plants with normal plants and their conidial concentrations was not feasible. Regression analysis revealed a negative relationship between eclosion rate and the logarithm of fungal conidial concentration (Fig 2A).

**Table 1:** Effects of IfB01 strain on MEAM1 eclosion rate (fungal treatment experiment 1).

Conidial concentration (×10^6^ spores/mL)	TY-plants /normal plants								
	3/3			2/6			1/5		
	2^nd^ nymphs	adults	eclosion rate*	2^nd^ nymphs	adults	Eclosion rate*	2^nd^ nymphs	adults	eclosion rate*
100.0	1800	204	11.33±0.58d	1800	222	12.33±0.58d	1800	174	9.67±0.58d
10.0	1800	426	23.67±1.53c	1800	528	29.33±1.15c	1800	444	24.67±0.58c
1.0	1800	753	41.83±2.93b	1800	786	43.67±2.31b	1800	819	45.50±0.87b
0 (CK)	1800	1488	82.67±4.62a	1800	1590	88.33±2.52a	1800	1506	83.67±2.31a

In this experiment involving potted tomato in cages, the conidial suspension was prepared using an IfB01 strain of *I*. *fumosorosea*. The TY-plants / normal plants in each cage were 3/3, 2/6 or 1/5, respectively. The MEAM1 2^nd^ instar nymphs were 300 in all the TY-plants in each cage. The MEAM1 2^nd^ instar nymphs were treated with conidial suspension through leaf immersion. The control (CK) was treated with 0.02% Tween 80 solution. The experiments were performed in triplicate and each experiment was repeated twice.

* The numbers labeled with different letters indicate significant differences (p < 0.05) by DMRT.

**Table 2:** ANOVA of eclosion rates of MEAM1.

Resource	Squariance	df	Mean square	F	*P*	Sig.
Calibrated model	27630.722	11	2511.884	144.453	0.000	*
Intercept	61669.444	1	61669.444	3546.486	0.000	*
Conidial concentration	27489.889	3	9163.296	526.963	0.000	*
TY-plants /normal plants	80.014	2	40.007	2.301	0.122	
Conidial concentration × TY-plants /normal plants	60.819	6	10.137	0.583	0.740	
Errors	417.333	24	17.389			
Total	89717.500	36				
Calibrated total	28048.056	35				

* Significant difference (p < 0.05).

### Effect of *I*. *fumosorosea* on TY-plants

Based on the results of fungal treatment experiment 1, the incidence of TY-plants (ITYP) on MEAM1 post-eclosion days 3 and 8 were significantly decreased (p < 0.05) depending on the fungal conidial dose after the 2^nd^ instar nymphs were treated with *I*. *fumosorosea* IfB01 strain (Table 3). ANOVA indicated significantly different (p < 0.01) ITYPs with various concentrations of fungal conidia and the TY-plants /normal plants. However, the differences in exposure to the conidial concentrations of TY- and normal plants were insignificant (Table 4). These results suggested that the conidial concentration of IfB01 strain and the TY-plants/normal plants showed a significant effect on ITYPs. However, the interaction of conidial concentrations with TY-plants /normal plants was not observed. Further regression analysis showed a negative relationship between eclosion rate and the logarithm of fungal conidial concentration (Fig 2A).

**Table 3:** Effects of IfB01 strain on ITYP in fungal treatment experiment 1.

	Conidial concentration (×10^6^ spores/mL)	TY-plants /normal plants								
		3/3			2/6			1/5		
		Total plants	TY-plants	ITYP (%)*	Total plants	TY-plants	ITYP (%)*	Total plants	TY-plants	ITYP (%)*	
Post-eclosion 3d	100	18	2	11.11±5.56d	36	10	27.78±2.78c	30	10	33.33±6.67c	
	10	18	5	27.78±5.55c	36	19	52.78±2.78b	30	14	46.67±3.33b	
	1	18	9	50.00±9.62b	36	21	58.33±0.00b	30	20	66.67±6.67a	
	0 (CK)	18	11	61.11±5.57a	36	27	75.00±4.81a	30	22	73.33±3.33a	
Post-eclosion 8d	100	18	8	44.44±5.56c	36	17	47.22±2.78c	30	15	50.00±5.77c	
	10	18	10	55.56±5.56b	36	25	69.44±5.56b	30	19	63.33±3.33b	
	1	18	11	61.11±5.56b	36	24	66.67±4.81b	30	21	70.00±5.77b	
	0 (CK)	18	14	77.78±5.55a	36	34	94.44±5.56a	30	25	83.33±3.33a	

In this study of pot-grown tomatoes in cage, the numbers of TY-plants / normal plants in each cage were respectively 3/3, 2/6 or 1/5. The original numbers of normal tomato plants in each cage are indicated. TY-plants are the newly *TYLCV*-affected plants excluding the original TY-plants treated with fungal conidial suspensions. The experiment was performed in triplicate, and repeated twice.

* The numbers labeled with different letters indicate significant difference (p < 0.05) by DMRT test.

**Table 4:** ANOVA of the incidence of TY-plants (ITYP).

Resource	Squariance	df	Mean square	F	*P*	Sig.
**ITYP at post-eclosion d 3 (%)**						
Calibrated model	13245.016	11	1204.092	14.382	0.000	*
Intercept	85230.910	1	85230.910	1018.039	0.000	*
Conidial concentration	10661.968	3	3553.989	42.451	0.000	*
TY-plants /normal plants	2254.400	2	1127.200	13.464	0.000	*
Conidial concentration × TY-plants /normal plants	328.648	6	54.775	0.654	0.687	
Errors	2009.296	24	83.721			
Total	100485.222	36				
Calibrated total	15254.313	35				
**ITYP at post-eclosion d 8 (%)**						
Calibrated model	3310.033	11	300.912	4.193	0.002	*
Intercept	134649.856	1	134649.856	1876.048	0.000	*
Conidial concentration	2566.445	3	855.482	11.919	0.000	*
TY-plants /normal plants	574.079	2	287.040	3.999	0.032	*
Conidial concentration × TY-plants /normal plants	169.509	6	28.251	0.394	0.876	
Errors	1722.556	24	71.773			
Total	139682.445	36				
Calibrated total	5032.588	35				

* Significant difference (p < 0.05).

**Fig 3:**
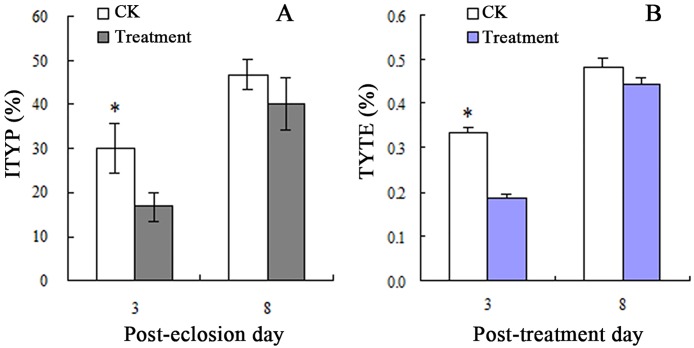
ITYPs (A) and TYTE (B) in fungal treatment experiment 2. * indicating significant differences between the two means by T-test. In this pot-cultured tomato in cage, the conidial suspension contained the IfB01 strain of *I*. *fumosorosea*. The MEAM1 2^nd^ instar nymphs in TY-plants were exposed to a conidial concentration of 10×10^6^ spores/mL via leaf immersion. After eclosion, each of the normal tomato plants was infected with three adults. The control was treated with healthy MEAM1 adults.

In the fungal treatment (experiment) 2, in which each normal plant was exposed to 3 adults, the fungus infected MEAM1 showed lower ITYPs (Fig 3). The T-test indicated that on post-treatment day 3, the ITYP in the treatment group was 16.67%, which was significantly (P < 0.05) lower than the ITYP of the control group (30%). However, ITYPs after the treatment (40.00%) and control (46.67%) on post-treatment day 8 were not significant. Obviously, the increased ITYP values on post-treatment day 8 were not attributed to transmission because all the MEAM1 adults were removed on post-treatment day 3. Probably, *TYLCV* levels were inadequate in plants on the post-treatment day 3. However, after 5 days of proliferation, the *TYLCV* was substantial and detectable.

### Effects of *I*. *fumosorosea* on TYLCV transmission by each MEAM1 adult (TYTE)

**Fig 4:**
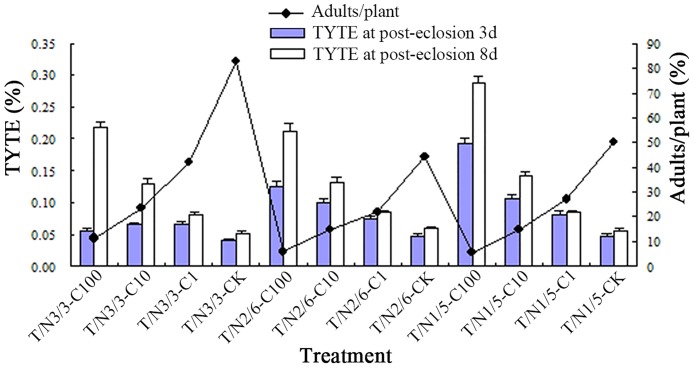
*TYLCV* transmission efficiency of each MEAM1 adult in the fungal treatment experiment 1. T/N 3/3-C100: TY-plants/ Normal plants = 3/3 with fungal conidia 100.0×10^6^ spores/mL; others were designated similarly.

The TYTEs were evaluated according to the fungal treatment experiment 1 (Fig 4). The TYTE values were closely affected by the fungal concentrations, the transmission times (days of post-eclosion), the number of normal plants (TY-plants/Normal plants, T/N) and insect numbers on each normal plant (adults/plant). Totally, the number of TYTEs increased with the transmission duration and normal plant growth. However, the number of TYTEs decreased with reduced fungal concentrations and increase in the number of adults/plant. Obviously, the higher number of adults in each plant significant decreased the TYTEs within the transmission capacity of each adult. For example, treatment of T/N2/6-C100 with 6 adults per plant yielded a 0.13% TYTE, which was remarkably larger than 0.05% TYTE detected with T/N2/6-CK involving 44 adults per plant on post-treatment day 3 (Fig 4). Nevertheless, the significantly lower ITYP of 27.78% related to 75.00% control was recorded in the same treatment (Table 3), suggesting that the TYTEs in fungal treatment experiment 1 does not reflect the true viral transmission capacity of insects.

In an additional experiment, in which every normal plant was only introduced to three adults, the TYTE was significantly reduced with the fungal treatment compared with the control on post-treatment day 3 (Fig 3B). Obviously, this result was contrary to the data obtained from the first experiment. Therefore, the actual TYTEs were masked by the large numbers of adults on each plant in experiment 1. The second experiment carrying relatively fewer numbers (3 adults/plant) offered more realistic TYTE values.

### Effects of *I*. *fumosorosea* on the incidence of TY-adults (ITYA)

**Fig 5:**
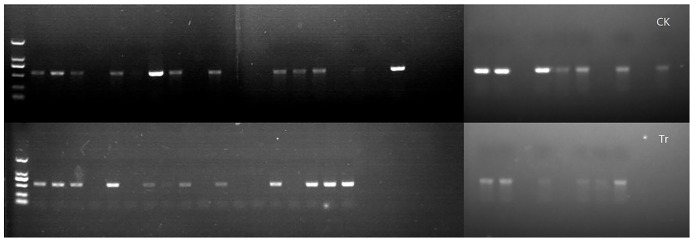
*TYLCV* PCR test of MEAM1 adults in fungal treatment experiment 2. CK: Control group, Tr: treatment group. The *AC2* gene fragment (322 bp) of *TYLCV* in each MEAM1 adult at post-eclosion day 3 was detected. The treatment group included the 2^nd^ instar nymphs of MEAM1 infesting TY-plant in the cage treated with the conidial suspension of 10×10^6^ spores/mL of IfB01 strain of *I*. *fumosorosea*. The control group was treated only with 0.02% Tween 80 solution. Thirty adults were detected.

The *TYLCV AC2* genes of MEAM1 adults on post-eclosion day 3 were amplified using PCR in the fungal experiment 2. The results indicated that *TYLCV*s were found in 17/30 adults in the treatment group and 18/30 adults in the control group (Fig 5), which indicated that the ITYAs in the treatment and control groups were not statistically different.

### Effects of *I*. *fumosorosea* on accumulation of TYLCV in individual MEAM1 adult

**Fig 6:**
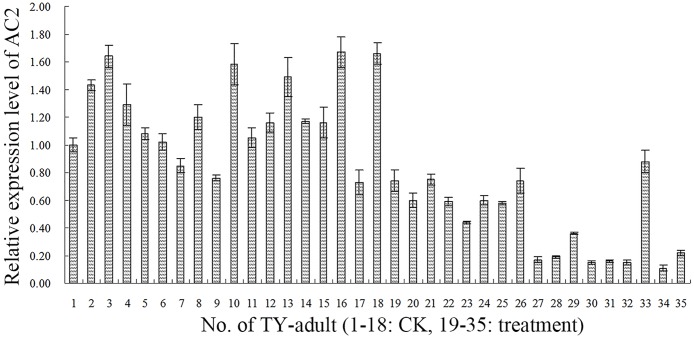
Relative expression of *TYLCV AC2* gene in the adults tested in fungal treatment experiment 2. The expression of the *AC2* (322 bp) in *TYLCV* of each TY-adult on post-eclosion day 3 was detected by qPCR. Treatment group comprised 2^nd^ instar nymphs of MEAM1 on TY-plant in cage treated with a conidial suspension of 10×10^6^ spores/mL from IfB01 strain of *I*. *fumosorosea*. The control group was treated only with 0.02% Tween 80 solution. Thirty adults were detected.

The cumulative *TYLCV* levels in individual MEAM1 adults were evaluated by qPCR amplification of *AC2* gene in the *TYLCV*-carrying adults of the fungal treatment 2. The results indicated that the cumulative levels of *TYLCV* in each adult in the treatment group were apparently lower than in the control group (Fig 6) suggesting that the cumulative levels of *TYLCV* in TY-adults were likely reduced after the MEAM1 nymphs were infected by *I*. *fumosorosea*.

## Discussion

The incidence and epidemic of insect-transmitted pathogenic viral disease is influenced by complex factors associated with the host plant, virus, vector and environment. Recognition of the interaction between host, virus, insect vector, and entomopathogenic fungus is essential to enhance our knowledge of plant viral disease and insect pests. In this study, we first focused on the relationship between tomato, *TYLCV*, MEAM1, and *I*. *fumosorosea*. In order to determine the role of fungal infection in the insect in *TYLCV* transmission by MEAM1, we conducted fungal experiment 1 to survey the variation in the incidence of TY-plants at different dosages of fungal treatment in nymphs and the incidence rates in TY-plants / normal plants. The results indicated that the incidence of TY-plants (ITYP) was substantively decreased after the plant carrying nymphs was treated with *I*. *fumosorosea*, suggesting that *I*. *fumosorosea* infection decreased the eclosion of MEAM1, and reduced the ITYPs.

We investigated the *TYLCV* transmission efficiencies of each adult (TYTEs) to evaluate the relationship between ITYPs and MEAM1 capacity of *TYLCV* transmission. However, we found that TYTEs declined with reduced concentrations of fungal treatment in the first experiment. Further analysis revealed that the excessive number of adults in each plant significantly reduced the TYTEs and was within the limits of every adult. Apparently, the actual TYTEs were masked by the higher numbers of adults in each plant. Therefore, the TYTE failed to reflect the insect’s true viral transmission capacity.

Therefore, the fungal experiment 2 introduced three MEAM1 adults on each plant. As expected, significantly decreased ITYPs and TYTEs were observed with fungal treatment (10×10^6^ spores/mL) compared with the control on post-treatment day 3. Apparently, the different ITYPs were related to fungal infection but not to reduced eclosion. Further, the TYTE values in the second experiment were more realistic.

Furthermore, we inspected the *TYLCV* in each MEAM1 adult in the second experiment. The results indicated that ITYAs in the treatment and control groups were not statistically different. However, the apparently lower *TYLCV* accumulation of TY-adults in the treatment group than in the control group was validated by further qPCR tests on post-treatment day 3. Thus, it is reasonable that the lower ITYPs in fungal treatment relate to smaller *TYLCV* levels in the MEAM1 adults and therefore, smaller *TYLCV* titers in the tomato plants. As a result, the virus levels are undetectable. However, after 5 days (on post-treatment day 8), the virus proliferation is significant and substantial for PCR detection.

ITYPs are attributed to viral transmission by TY-adults. However, in this study, after MEAM1 nymphs are inoculated with *I*. *fumosorosea*, most of the nymphs are infected by fungus and finally die from fungal disease. However, the other nymphs uninfected by the fungus eventually develop into adults, which are the main reservoir of TY-adults. Additional TY-adult resource includes infected nymphs, which develop into adults. Therefore, we investigated the gene fragments of fungal rDNA-ITS in MEAM1 adults by PCR, and unfortunately, the fungus was not found (data unpublished). This result suggests that fungus was not found in surviving MEAM1 adults. However, the limited number of few fungal cells in the adults was undetectable through PCR.

The lower levels of *TYLCV* in MEAM1 adults might be related to specific substances produced by the fungi, which decrease feeding or interfere with *TYLCV* replication and transcription. In fact, entomopathogenic fungi usually produce a few compounds and specific proteins that inhibit other micro-organisms in the host [30,31,32]. For example, *Metarhizium anisopliae* secretes destruxins to suppress host immunity and viral activity [33]. Specific materials isolated from *I*. *fumosorosea* inhibited the virus, suggesting possible control of *TYLCV* by reducing the viral titers in MEAM1. Therefore, the management of insect-transmitted plant viruses is facilitated via control of viral levels in insect vectors.

The reduction of *TYLCV* in MEAM1 adults is probably related to host plants and endophytic fungal entomopathogens. Several reports of endophytic fungal entomopathogens were related to *Beauveria bassiana*, *Metarhizium anisopliae*, *Purpureocillium lilacinum* (Formerly *Paecilomyces lilacinus*) and *Lecanicillium lecanii*, which affect insect growth, development and reproduction [34,35,36,37]. If *Isaria fumosorosea* was endophytic after inoculation on tomato plants, additional investigation is desirable.

In conclusion, ITYPs and TYTEs were reduced by MEAM1 nymphs treated *with I*. *fumosorosea* IfB01 strain. Fungal treatment of MEAM1 nymphs decreased the eclosion rates and the cumulative levels of *TYLCV* in MEAM1 TY-adults. Consequently, infection of nymphs with *I*. *fumosorosea* IfB01 strain eventually reduces the adult numbers and decreases the *TYLCV* transmission capacity of TY-adults. Our study provides new insight into interactions between host plant, phytopathogenic viruses, insect vectors and entomopathogenic fungi.

## Supporting Information

S1 Data The original data of experiments (supplementary materials). (XLS)
